# Supplementary material for: Effect of Drought Stress on Capsaicin and Antioxidant Contents in Pepper Genotypes at Reproductive Stage
Source: Plants (Basel). 2021 Jun 24;10(7):1286. doi: 10.3390/plants10071286 (PMC8309139; doi:10.3390/plants10071286)
Supplement: Supplementary file 1 [file plants-10-01286-s001.zip › plants-1234242-supplementary.pdf]

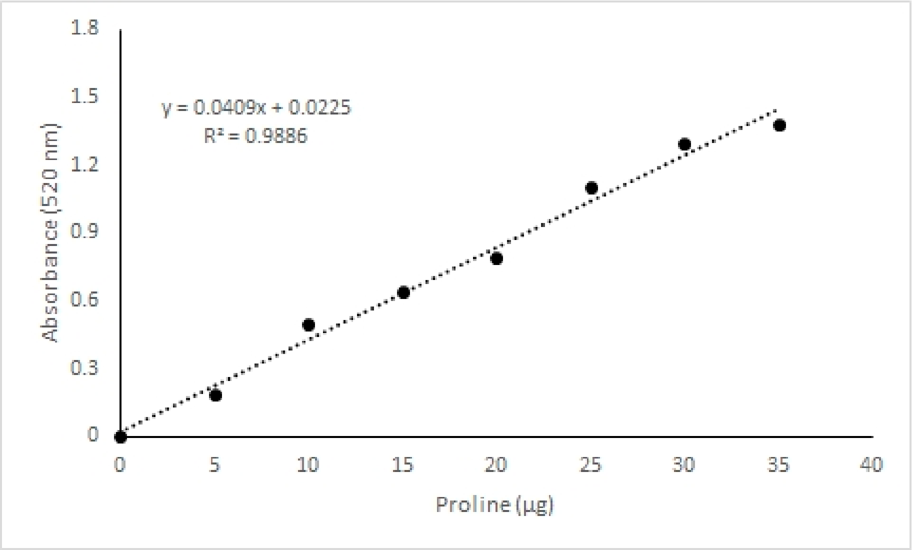

Supplementary Figure S1. Standard curve of proline estimation by ninhydrin method

Supplementary Table S1. Mean Square values obtained from Analysis of Variance (ANOVA). *Significant at 5% probability, **Significant at 1% probability. DF= water stress at flowering stage, DP = water stress at pod formation stage.

	SOV	Genotype	Error
Proline	Control	17.40**	0.02
	DF	37.68**	0.03
	DP	42.76**	0.05
Fresh wt of fruits	Control	43.529**	0.0301
	DF	17.276**	0.0105
	DP	16.787**	0.0091
Dry wt of fruits	Control	0.508**	0.0014
	DF	1.259**	0.0010
	DP	1.209**	0.0009
Fruits per plant	Control	3237.9**	17.3
	DF	9.36**	0.24
	DP	16.62**	0.18
Ascorbate peroxidase	Control	330.22**	17.05
	DF	451.96**	5.24
	DP	720.44**	40.48
Catalases	Control	501.29**	21.7
	DF	514.81**	6.78
	DP	298.19**	39.65
Guaiacol	Control	560.80**	8.16
	DF	2742.4**	10.18
	DP	11705**	82.9
Fruit shape index	Control	3.80**	0.02
	DF	3.82**	0.02
	DP	8.00**	0.04
Flower survival %	Control	7681.9**	145.9
	DF	10837**	124.3
	DP	15256**	128.8

Supplementary Table S2. Capsaicin contents in four pepper genotypes

		Scoville units	Scoville units	Scoville units
	Genotype	Control	D(F)	D(P)
Hot Peppr	Pusa juala	50000	50000	10000
	Ghotki	60000	60000	10000
	PPE-311	10	10	10
Sweet Pepper	Green wonder	10	10	10
